# The Epidemiology of Food Allergy in the Global Context

**DOI:** 10.3390/ijerph15092043

**Published:** 2018-09-18

**Authors:** Wenyin Loh, Mimi L. K. Tang

**Affiliations:** 1 Allergy and Immune Disorders, Murdoch Children’s Research Institute, Melbourne 3052, Australia; wenyin.loh@mcri.edu.au; 2 Allergy Service, Department of Paediatrics, KK Women’s and Children’s Hospital, Singapore 229899, Singapore; 3 Department of Allergy and Immunology, The Royal Children’s Hospital, Melbourne 3052, Australia; 4 Department of Paediatrics, University of Melbourne, Melbourne 3010, Australia

**Keywords:** food allergy, prevalence, time trends, Asia, Westernized countries

## Abstract

There is a lack of high-quality evidence based on the gold standard of oral food challenges to determine food allergy prevalence. Nevertheless, studies using surrogate measures of food allergy, such as health service utilization and clinical history, together with allergen-specific immunoglobulin E (sIgE), provide compelling data that the prevalence of food allergy is increasing in both Western and developing countries. In Western countries, challenge-diagnosed food allergy has been reported to be as high as 10%, with the greatest prevalence noted among younger children. There is also growing evidence of increasing prevalence in developing countries, with rates of challenge-diagnosed food allergy in China and Africa reported to be similar to that in Western countries. An interesting observation is that children of East Asian or African descent born in a Western environment are at higher risk of food allergy compared to Caucasian children; this intriguing finding emphasizes the importance of genome-environment interactions and forecasts future increases in food allergy in Asia and Africa as economic growth continues in these regions. While cow’s milk and egg allergy are two of the most common food allergies in most countries, diverse patterns of food allergy can be observed in individual geographic regions determined by each country’s feeding patterns. More robust studies investigating food allergy prevalence, particularly in Asia and the developing world, are necessary to understand the extent of the food allergy problem and identify preventive strategies to cope with the potential increase in these regions.

## 1. Introduction

Food allergy is increasingly recognized as a growing public health burden and has been referred to as the “second wave” of the allergy epidemic, following asthma [1]. Current evidence suggests that food allergies are common, affecting up to 10% of infants in some countries [2], and have been increasing in prevalence in the last few decades. These increases in prevalence have preferentially affected industrialized regions, although there is now also growing evidence of increasing prevalence in rapidly developing countries commensurate with rising economic growth [3].

Accurate determination of food allergy prevalence confirmed by the gold standard of food challenge is resource intensive, which limits the availability of quality data, as emphasized in a recent international survey [4]. In a survey of 83 World Allergy Organization member countries and six non-member countries, over half (*n* = 51) had no data on food allergy prevalence, while a quarter (*n* = 23) had data based on patient/parent report, and only 10% (*n* = 9) had food allergy prevalence data based on oral food challenges (OFCs). Paucity of robust data is further compounded by the inconsistent definitions and methodologies used in the various studies. Currently, the majority of available data is based on self-reporting, which generally overestimates food allergy prevalence by a factor of three to four [5]. This may be due to patients/parents mistaking other adverse reactions to food (e.g., food poisoning, enzyme deficiencies, contact dermatitis, or food aversions) for food allergy. Wide variability in reported food allergy rates within a single country when both OFC and self-reported data has been available confirms the inaccuracy of self-reported food allergy as a measure of true prevalence. For example, prevalence of parent-reported food reactions was 14.5% among siblings of 1570 German infants enrolled in the EuroPrevall study [6], whereas a separate study reported OFC confirmed food allergy in 31 of 739 German children (4.2%) [7]. One surrogate measure of food allergy which has been suggested to provide greater accuracy in determining food allergy prevalence is the presence of a clinical history of reaction to the food in combination with a positive allergen-specific immunoglobulin E (sIgE) or skin prick test. However, it is important to note that most sensitized individuals are able to tolerate the food without reaction, so this approach may still overestimate the true prevalence if the history of reaction is based on self-report [2]. Food anaphylaxis admissions or presentations to the emergency department have also been used as surrogate measures, but are subject to issues of variable definitions and coding. In particular, multiple episodes in a single individual need to be distinguished from multiple individuals with a single presentation when using these surrogate measures to determine prevalence.

An important consideration when evaluating time trends in food allergy prevalence is that the point prevalence is determined by the combined impact of new incident cases and resolution of existing cases. Hence, although most countries have reported an increase in food allergy prevalence during the last few decades, it is not clear whether this increase relates to an increase in the number of newly diagnosed cases (incidence) or a trend towards food allergies being more persistent in recent decades [8]. In the absence of an effective cure, and with many food allergies persisting throughout life, it is expected that prevalence will increase even if the incidence remains the same.

## 2. Food Allergy Prevalence in Western Countries

In children less than 5 years of age, prevalence of challenge-proven food allergy has been reported to be 4% in the UK [9], 3.6% in Denmark [10], 6.8% in Norway [11] and more than 10% at age 12 months and around 4% at age 4 years in Australia [2,12]. In the Melbourne-based HealthNuts study, prevalence of peanut allergy at 12 months was 3.0% (95% CI, 2.4–3.8), egg allergy was 8.9% (95% CI, 7.8–10.0) and sesame allergy was 0.8% (95% CI, 0.5–1.1) [2]. At the four-year follow-up, peanut allergy prevalence was 1.9% (95% CI, 1.6–2.3), egg allergy was 1.2% (95% CI, 0.9–1.6) and sesame allergy was 0.4% (95% CI, 0.3–0.6) [11]. A systematic review that included 42 studies published in Europe between 2000 and 2012 [13] reported the point prevalence of challenge-confirmed food allergy to cow’s milk, egg, wheat, soy, peanut, tree nuts, fish and shellfish to be 0.6% (95% CI, 0.5–0.8), 0.2% (0.2–0.3), 0.1% (0.01–0.2), 0.3% (0.1–0.4), 0.2% (0.2–0.3), 0.5% (0.08–0.8), 0.1% (0.02–0.2), and 0.1% (0.06–0.3), respectively. In the same systematic review, the overall pooled estimates for self-reported lifetime prevalence of allergy to cow’s milk, egg, wheat, soy, peanut, tree nuts, fish and shellfish were 6.0% (95% CI, 5.7–6.4), 2.5% (2.3–2.7), 3.6% (3.0–4.2), 0.4% (0.3–0.6), 1.3% (1.2–1.5), 2.2% (1.8–2.5), and 1.3% (0.9–1.7), respectively. These results highlight the very poor correlation between self-reported and challenge-confirmed food allergy. This systematic review also highlighted the significant heterogeneity and wide variation in participation rates between studies, with some participation rates as low as 17%. In the U.S., an electronic survey of self-reported food allergy in children less than 18 years of age (*n* = 38,480) revealed a prevalence of 8.0% (95% CI, 7.6–8.3) [14], which is consistent with the findings for Europe reported in the systematic review.

Studies assessing food allergy prevalence among older children and adults are even scarcer. In one of the few studies that used OFCs to confirm diagnosis, the prevalence of food allergy among 11 and 15-year-olds in the UK, as determined by either OFC or a history of a clinical reaction with a positive skin prick test, response was 2.3% (18 of 775) in the 11-year-old cohort and 2.3% (17 of 757) in the 15-year-old cohort [15]. A German study that evaluated children across all ages (0–17 years, mean age 9.2 years) reported a prevalence of OFC-confirmed food allergy of 4.2% [7], and a study from Turkey reported a rate of 0.15% among adolescents between 11 and 15 years of age [16]. In Australia, a country with one of the highest rates of food allergy, a recent study evaluating 5016 adolescents aged 10 to 14 years reported an OFC-confirmed food allergy prevalence of 4.5% (95% CI, 3.9–5.1) [17].

## 3. Time Trends in Food Allergy Prevalence in Western Countries

Ideally, change in prevalence over time should be evaluated using the same methodology in the same population at sequential times. Most countries have reported an increase in food allergy prevalence over the last decade. There are no studies in Western countries with repeated measures of challenge-proven food allergy, and the majority of studies have instead evaluated changes over time using hospital anaphylaxis admission rates or increasing health care burden as a surrogate measure for food allergy prevalence. In the UK [18], admission rates for anaphylaxis due to a food trigger rose from 1.2 to 2.4 per 100,000 between 1998 and 2012. In Australia, admissions for anaphylaxis caused by food showed an average annual increase of 13.2% between 1994 and 2005. The highest rate of increase was reported among those aged 0 to 4 years with a 5.5-fold increase demonstrated over the 12-year study period [19]. Furthermore, it was noted that admission rates were increasing most quickly for peanut- and crustacean-induced anaphylaxis (a greater than three-fold increase in five years), whereas rates of milk and egg allergy had only increased ~1.5 fold in the same period [20]. A subsequent study using the same methodology to examine Australian hospital morbidity data showed that food-related anaphylaxis admission rates have continued to increase from 5.6 to 8.2 per 100,000 between 2005 and 2012 [21]. Again, the highest rates occurred in children aged zero to four years. A similar increase in hospitalizations for allergic reactions between 1999 and 2011 has been reported in Finland and Sweden [22], while countries that reported stable prevalence of food allergy include Canada [23,24] and the U.S. [25]. A U.S. study using a nationwide, computer-assisted survey technology and sampling system reported no increase in self-reported peanut and tree nut allergy between 1997 and 2008 (1.4% in 2008 compared with 1.2% in 2002 and 1.4% in 1997) [25]. There have been no reports of decreasing food allergy prevalence in Western countries.

## 4. Food Allergy Prevalence Outside Europe, the US and Australia

In other parts of the world, reported prevalence of challenge-proven food allergy has varied widely, and until recently it was perceived that food allergy was uncommon in the developing world. However, prevalence of challenge-proven food allergy is reported to range from 1% among children aged three to seven years in Thailand [26] to 3.8% and 7.7% of one- and two-year-old children in China respectively [27,28]. Furthermore, two recent studies of challenge-proven food allergy in South Africa reported prevalence rates ranging from 2.5% in an unselected population of children to 40% in children with moderate to severe atopic dermatitis [29,30].

Other studies using surrogate measures to identify food allergy have found similar rates of food allergy in Asian and African countries. A study in Ghana using a combination of food allergy symptoms and sIgE reported a prevalence of 11% [31]. Self-reported and questionnaire-based food allergy rates in other East Asian countries, including South Korea [32], Japan [33], Hong Kong [34,35] and Taiwan [36], have been reported to range from 3.4 to 7.0%. High rates of food sensitization and self-reported food allergy ranging from 5–19% have been noted in parts of Africa [31,37,38]. In contrast, using clinical symptoms and positive sIgE to detect food allergy, the prevalence of food allergy in South India was only 1.2% amongst 11,000 randomly selected adults aged between 20 and 54 years [39]. Interestingly, higher rates of sensitization were noted in Bangalore (34.0% of men, 29.5% of women) compared to Mysore (19% of men, 18.6% of women). This is thought to reflect the westernization of Bangalore (also commonly referred to as the Silicon Valley of India) in recent years.

There is limited data on changes in prevalence over time in developing countries. A study in China reported a doubling in prevalence of OFC-diagnosed food allergy amongst 0–24-month-old infants recruited from well-baby checks over a period of 10 years from 3.5% in 1999 to 7.7% in 2009 (*p* = 0.02) [28].

## 5. Risk Factors for Food Allergy

Despite the limitations of existing prevalence data, these studies provide valuable information regarding the extent of the problem and risk factors that contribute to rising prevalence. Food allergy, as with most chronic disorders, results from complex interactions of genetic and environmental factors in early life. Both modifiable and non-modifiable early life risk factors have now been identified, including male sex, ethnicity, genetics, microbial exposure (improved hygiene, antibiotic use, dog exposure), allergen exposure (timing and route of exposure, antacid use) and vitamin D insufficiency [40,41]. It appears a shift towards an urbanized lifestyle, either as a result of rising economic growth or migration, is associated with development of food allergy. Studies on migration, in particular, highlight the important interplay of ethnicity and the environment.

In Australia, it was noted that 12-month-old infants with parents of East Asian ethnicity had a three-fold higher risk of food allergy compared with infants of non-East Asian descent [42]. Furthermore, children with Asian mothers who were born in Asia and later migrated to Australia had a lower risk of nut allergy (adjusted odds ratio, 0.1; 95% CI 0.03–0.31) than children with Asian mothers born in Australia. These findings suggest that genetic factors associated with Asian heritage may confer an increased risk of food allergy in infants exposed to a Western environment in early life. In this regard, it has been reported that eight different filaggrin (FLG) mutations account for 80% of FLG mutations in Singapore, a country with a large Chinese population, compared to only two dominant FLG null mutations noted in European countries [43]. This observation, along with one made in the HealthNuts study, that atopic eczema was more likely in infants of East Asian descent, reinforces the importance of genome-environment interactions [44]. Similarly, differences in food allergy prevalence related to ethnicity have been noted in New Zealand, where Pacific Islanders have higher anaphylaxis admission rates compared to other ethnic groups [45], and in the U.S., where Black American children have higher rates of food allergy than White Americans [46]. Extrapolating from this concept, food allergy prevalence may be expected to increase in Asia and Africa as there is increased urbanization and adoption of a Westernized lifestyle.

## 6. Global Variation in Common Triggers of Food Allergy and Food-Induced Anaphylaxis

While cow’s milk, egg, peanut, tree nuts, fish, shellfish, wheat and soy account for the majority of allergic reactions in most countries, differing geographical locations and feeding patterns exert a significant influence on the list of common allergens in each particular region. Furthermore, the types of foods responsible for causing reactions differ depending on the age group, with allergy to cow’s milk and egg more common among younger children and allergy to peanut, tree nuts, fish and shellfish more common in older children and adults [3,13].

Apart from cow’s milk and egg allergy, which are consistently amongst the most prevalent food allergies in children irrespective of geographic region, the patterns of food allergy across the U.S., Europe and Asia are otherwise quite different, reflecting the varied diets consumed in different countries. Fruit allergies due to cross-reactions with inhalant allergies are common in Europe [47]. In pollen-related food allergies, primary sensitization to tree pollen results in cross-reaction to homologous allergen structures present in fruits. In Northern Thailand, the top five foods that cause food allergy in children are shrimp, cow’s milk, fish, egg and ant eggs, with shrimp being the most common food trigger [26], whereas in India chickpea is a major food allergen, reflective of the typical South Asian diet that frequently includes legumes [48]. Indeed, food triggers responsible for severe allergic reactions in Asia include common, and in some instances unique, components of the Asian diet: fish, shellfish, buckwheat, bird’s nest and royal jelly.

Shellfish is the leading cause of anaphylaxis in adults and older children in Hong Kong, Taiwan, Singapore and Thailand [49,50,51,52]. The prevalence rates among adolescents are reported to be 5.12% and 5.23% in the Philippines and Singapore, respectively [53], compared to 0.7% in the U.S. [54]. On the other hand, buckwheat is one of the main causes of anaphylaxis in South Korea and Japan, likely because of the frequent consumption of buckwheat noodles in these countries [55,56]. Other unique food allergens include bird’s nest from swiftlets causing allergic reactions in Singapore [57] and Malaysia [58], where it is considered a delicacy, as well as royal jelly in Hong Kong [59].

Interestingly, although peanut was previously not a common cause of anaphylaxis in Asia, this appears to be changing with adoption of a Westernized lifestyle. A recent study of Singaporean children reported that peanut had emerged as the most common food trigger of anaphylaxis [60], whereas a similar study conducted 15 years ago reported bird’s nest as the most common trigger of anaphylaxis, while peanut and tree nuts were not identified as triggers at all [61]. The authors postulated that the changing pattern of food anaphylaxis in Singaporean children could be due to more children now consuming processed peanut butter as the first exposure to peanut, compared to the first exposure being to boiled peanuts in soup or porridge in previous years.

## 7. Conclusions

Food allergy is a growing health concern, with increasing prevalence noted not just in Westernized countries but also in developing countries. More robust studies using standardized methodologies and objective methods of assessment are necessary for accurate detection of food allergy in order to better understand the true extent of the problem and its impact on health services. It is clear that for the prevalence of food allergy to stabilize or fall, strategies to hasten disease resolution and reduce disease incidence are required. While there is presently no cure for food allergy, with management focused on allergen avoidance and prompt treatment of allergic reactions, there is ongoing intense research in the area of food allergen immunotherapy as a means of inducing tolerance. Oral immunotherapy (OIT), in particular, has been shown to be effective at inducing desensitization [62]. However, the limited ability of OIT to induce tolerance and the associated risks of adverse reactions suggest that further investigation is required before OIT can be recommended in clinical practice. Disease incidence reduction can only be possible with identification of modifiable risk factors. Of particular interest is the changing pattern of food allergy in Asia and the developing world, not just because of the dramatic increase that can be expected given the genetic predisposition combined with ongoing industrialization, but also because tailored preventive strategies may be required for different geographic regions.
